# Intergenerational Cooperation at Work to Promote Inclusion and Healthy Participation of Older Workers in the Foodservice Sector: Protocol for a Qualitative Study

**DOI:** 10.2196/79012

**Published:** 2025-12-01

**Authors:** Caroline Chevrier, Alexandra Lecours, Marie-Michèle Lord

**Affiliations:** 1 Occupational Therapy Department Université du Québec à Trois-Rivières Trois-Rivieres, QC Canada; 2 Interdisciplinary Research Center on Rehabilitation and Social Integration (Cirris) Québec, QC Canada; 3 Occupational Therapy Department Université du Québec à Trois-Rivières Drummondville, QC Canada; 4 Centre for Research and Expertise in Social Gerontology (CREGÉS) Côte-Saint-Luc, QC Canada

**Keywords:** intergenerational cooperation at work, older workers, healthy participation, inclusion, foodservice sector, photo-elicitation, focus groups, TRIAGE method, Technique for Research of Information by Animation of a Group of Experts

## Abstract

**Background:**

Increasing intergenerational coexistence in the job market may heighten the risk of intergenerational conflicts, which can negatively impact the inclusion and healthy participation of older workers. The foodservice sector is characterized by significant intergenerational coexistence, reinforced by the presence of student workers. According to the literature, practices promoting intergenerational cooperation can reduce the risk of conflict and contribute to a healthier work climate.

**Objective:**

The main objective of this research project is to propose a prioritized list of intergenerational cooperation practices to promote the inclusion and healthy participation of older workers in the foodservice sector.

**Methods:**

This project will be carried out in 2 stages. The first stage will use a descriptive interpretative design based on the photo-elicitation method to identify intergenerational cooperation practices from the perspective of older workers. The second stage will use the Technique for Research of Information by Animation of a Group of Experts method to obtain a group consensus among professionals, managers, and researchers to prioritize the identified practices.

**Results:**

The first stage of this project is underway. The study has been funded by the Quebec Network for Research on Aging since September 2024. Participant recruitment began at the end of January 2025, following ethics committee approval, and ended in May 2025. In total, 12 participants were recruited for stage 1. The data collected are currently being analyzed, and the results are scheduled to be submitted for publication in 2026.

**Conclusions:**

On a theoretical level, this project will propose a list of practices promoting intergenerational cooperation at work in the foodservice sector, giving a voice to older workers to better understand their perspectives. This project will also offer a prioritization of those practices, based on the perspective of different actors (researchers, professionals, and managers of the foodservice sector). On a practical level, this knowledge will help the actors of the foodservice sector to integrate these practices into their organizations to promote the inclusion and healthy participation of older workers.

**International Registered Report Identifier (IRRID):**

DERR1-10.2196/79012

## Introduction

### Background

Older workers, aged 55 years and older, are an increasing part of the workforce in Canada [1,2]. For example, in the Canadian province of Quebec, more than 20% of workers are aged 55 years or older [1]. The number of young workers has never been so low compared to older ones [1]. Nearly 75% of workers aged 55 to 59 years were active on the job market in 2021, compared to 55% in 2001 [3]. Given labor shortages, lower birth rates, the rise in life expectancy, and their growing proportion in the current workforce, older workers are in high demand to fill vacancies across all sectors [2,4]. The foodservice sector is one that has been particularly affected by the labor shortage in recent years [5]. Nearly 46.3% of businesses in the hospitality and foodservice sector forecast difficulties in recruiting staff in 2022 in Canada [6]. As a result, many restaurants have had to reduce their opening hours and implement strategies to attract and retain staff, such as increasing salaries [7,8]. These ongoing labor shortages, which have been growing for several years in the sector, have raised concerns about job insecurity [5] and continue to pose challenges today [9]. Hiring or helping older workers stay employed is seen as one of the solutions to address these shortages [2,10]. Although the foodservice sector is predominantly made up of young adults, it also includes 14% of older workers [9]. Those workers often work alongside colleagues aged between 15 and 24 years (44%) [11], illustrating a high degree of intergenerational coexistence at work, which can sometimes be challenging. How could this coexistence be eased in this sector? This is what we aim to explore in this study.

Depending on the legal or national context, up to 6 generations may coexist in today’s labor market: the Traditionalists, the Baby Boomers, and Generations X, Y, Z, and Alpha [12]. This intergenerational diversity can be beneficial on several levels, as organizations can benefit from a variety of perspectives [13]. However, this diversity can also increase the risk of intergenerational conflict, which can be defined as disagreement or tension between people of different generations [14,15]. Such conflicts may arise from various factors such as age-related prejudices [16,17], stereotyping or discrimination [18], perceived differences [19], conflicts of values, differences regarding work representation [20], communication and learning style differences [13], or even rivalry between members of different generations [15]. Some authors argue that these conflicts are rooted in real differences in values and perspectives, while others suggest that they may stem more from perceived differences. Despite government policies against discrimination in the workplace, ageism remains a persistent issue [21]. In the foodservice sector, which is predominantly made up of young workers, age discrimination is still observed in recruitment, training, and job retention [22,23].

Intergenerational conflicts can undermine the inclusion and healthy participation of older workers. Ageism and conflicts may lead to reduced job satisfaction, lower engagement, and diminished self-esteem among older employees, who may begin to question their professional competencies [14,24]. Such tensions may reduce collaboration between age groups [14], causing generational fragmentation that limits knowledge transfer, team cohesion, and support between colleagues, an important psychosocial factor. Increased psychosocial risks are associated with adverse outcomes such as absenteeism, injuries, and early retirement among older workers [25]. The foodservice sector is particularly exposed to these stressors, including irregular hours, high service pressure, demanding customer interactions, and cognitive overload [26,27]. Promoting intergenerational cooperation is seen as a promising strategy to mitigate the risk of intergenerational conflict and foster inclusion and healthier work environments [18,20,28].

Some intergenerational cooperation practices have been identified in the literature (eg, reverse mentoring, proactive intergenerational management model, and career legacy circle) [18,29]. However, such practices are not always well-established in workplaces, as organizations may fear not being able to manage significant changes, inadvertently favor one generation over another, or create tensions between generations if these practices are not properly implemented [18].

While there is some evidence on key guidelines for intergenerational cooperation in the workplace, the current literature lacks insights into concrete practices that various actors can implement to reduce intergenerational conflict and promote the inclusion and healthy participation of older workers. Although intergenerational cooperation has been studied across various fields, including nursing [30], education [31], transportation [32], and computer science [33], the foodservice sector remains largely unexplored in this regard. Many studies examine generational trends from the perspective of customers [34,35], but few have addressed the intergenerational experiences of workers themselves [36].

### General and Specific Objectives

The general objective of this research project is to propose a prioritized list of intergenerational cooperation practices to promote the inclusion and healthy participation of older workers in the foodservice sector. To achieve this, two specific objectives were set: (1) to identify individual, collective, organizational, and societal practices that foster intergenerational cooperation at work (ICW) and (2) to prioritize these practices for implementation in the foodservice sector.

### Conceptual Framework

#### Intergenerational Cooperation at Work

This project is based on the concept of ICW. Given that the existing literature provides limited concrete definitions to better understand and apply this concept in professional settings, we started by examining its meaning in the context of a previous study that specifically focused on its analysis. In that study, we proposed a definition of the concept [37]:

Intergenerational cooperation at work (ICW) can be defined as the achievement of social cohesion within intergenerational teams committed to a common goal, through their interaction, knowledge sharing, mutual support, understanding and acceptance of their differences, in a process of intentional change supported by the organization. ICW thus implies the inclusion of all generations in a healthy work environment that promotes intergenerational equity, trust, and respect. It requires the involvement of all actors, who must be convinced of its importance.

Although introducing ICW in an organization takes time, its potential benefits are significant. ICW can improve the organizational climate, employee motivation, satisfaction, and well-being [18]. For the organization, this translates into improved knowledge transfer, team efficiency, and productivity [38,39]. In addition, this conceptual work highlighted the role played by various actors in the workplace in enabling intergenerational cooperation, showing that the individual worker, the work team, and the organization must all contribute to this collective effort. Society may also have a role to play in implementing measures that support such cooperation and fostering the inclusion of older workers [40].

As part of this project, the definition of ICW will be shared with all participants involved in this project to ensure a common understanding of the concept. A shared understanding minimizes the risk of divergent interpretations that could affect the quality and consistency of the data collected. This definition will also serve as a framework for coding and interpreting the results, ensuring that the analysis is grounded in a consistent and shared interpretation of intergenerational cooperation.

#### Intergroup Contact Theory

This project is also grounded in Allport’s [41] theory of intergroup contact, which posits that bringing individuals from heterogeneous groups together can reduce stereotypes and prejudices by improving attitudes toward one another, provided 4 conditions are met [42]. First, group members must have equal status. Second, they must be able to cooperate, meaning there should be no competition between them. Third, they must work toward common goals. Finally, they must benefit from institutional and societal support that encourages their interaction. Applied to an intergenerational context, this theory suggests that pairing people from different generations at work and encouraging them to collaborate toward shared objectives could improve the work environment by reducing prejudice and stereotypes. In this way, their frequent interactions would help them understand and accept each other’s differences [42]. Given the challenges previously identified, such as intergenerational conflict and ageism in the workplace, Allport’s theory provides an appropriate framework to address these issues by fostering positive intergenerational contact. Furthermore, a study by Firzly et al [43] applied this theory in their research on ageism at work, knowledge sharing, and job satisfaction, exploring how age-related intergroup relations affect younger workers’ perceptions of older colleagues. This theory has also been applied by numerous researchers examining intergenerational relations in workplace settings, demonstrating that high-quality intergenerational contact can positively influence attitudes and help reduce ageism at work [44-46]. This conceptual framework will allow for a more comprehensive and nuanced analysis of the intergenerational cooperation practices identified in the study. In our analysis, we will pay particular attention to the conditions outlined by Allport (eg, presence of hierarchy, competition, shared goals, and organizational support) and how they may influence intergenerational dynamics.

## Methods

### Study Design

**Figure 1:**
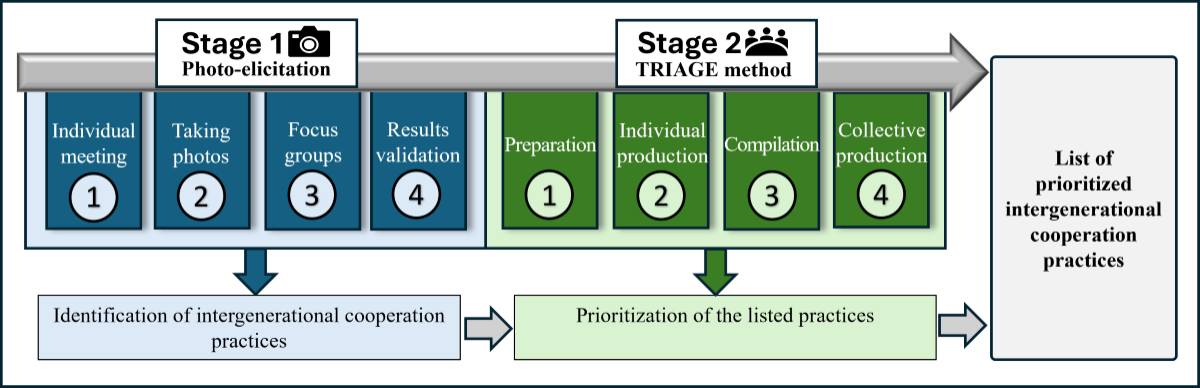
Stages of the research project. TRIAGE: Technique for Research of Information by Animation of a Group of Experts.

This research project will be carried out in 2 stages, as shown in Figure 1, which presents the process and how its components are interconnected. The first stage involves a photo-elicitation study to identify intergenerational cooperation practices, carried out in 4 steps. The second stage, based on the Technique for Research of Information by Animation of a Group of Experts (TRIAGE) method, aims to prioritize these practices in 4 phases as well.

### Stage 1: Identification of Practices That Promote or Hinder Intergenerational Cooperation

#### Design

A qualitative research design of the descriptive-interpretive type will be used to conduct this project, based on the photo-elicitation method. The descriptive-interpretive approach is used to gather participants’ perspectives in order to understand the meaning they assign to the phenomenon they experience [47]. The photo-elicitation method involves using photographs to enrich qualitative interviews [48]. First described by Collier [49], this method was originally used in anthropology and sociology before expanding into fields such as public health, education, and health care [48]. This approach will allow us to explore older workers’ perspectives on practices that promote or hinder ICW, providing access to images that evoke their personal narratives. This, in turn, makes it possible to observe elements that might be difficult to capture using more conventional methods [50,51]. The photo-elicitation method will also enable us to consider each participant’s unique perspective by allowing them to share their knowledge and experiential insights, thus fostering the participation and engagement of older workers in the research project [50]. The photo-elicitation method will be combined with focus groups to discuss the corpus of photographs. The project will also adopt an intersectoral approach, combining the experience of researchers from various fields, including health, social sciences, and engineering. Given that the concept of intergenerational cooperation spans various domains and can impact workers’ health, social relations at work, management practices, and even the use of technology, it is crucial to embrace this approach.

#### Participants

In line with evidence-based recommendations, we plan to conduct focus groups consisting of 6 participants each. The recommended number of participants for focus groups typically ranges from 5 to 12, allowing everyone sufficient time to share their views and ensuring diverse perspectives on the topic being explored [52]. The literature also suggests conducting at least 2 focus groups to ensure comprehensive coverage of the topic [52]. According to Guest et al [53], conducting 2 to 3 focus groups typically covers over 80% of the topics under study. Several aspects of our approach support early saturation [53]: the use of a semistructured interview guide, the clear and shared understanding of the research topic among participants, and the triangulation of data through participant-generated photographs. While the foodservice sector is diverse, our sample is relatively homogeneous in key aspects: all participants are older workers operating in the same sector, from the same region, and with similar levels of education. Moreover, the analytical framework is based on predefined categories (individual, collective, organizational, and societal practices), which allows for systematic and focused coding. However, in line with an iterative approach, the final number of participants will be determined by data saturation, which will be reached when the most significant elements related to our research topic have been covered during the focus groups [53].

Recruitment is ongoing. Participants are selected based on their interest in the study and meeting the eligibility criteria. They could come from both the commercial and noncommercial foodservice sectors. The commercial sector refers to establishments where food sales are the primary objective (eg, restaurants), while the noncommercial sector includes organizations where foodservice supports another main mission, such as long-term care facilities. The eligibility criteria are (1) being at least 55 years of age, (2) working in the foodservice sector, and (3) working with colleagues younger than 25 years of age.

This last criterion reflects our desire to ensure a workplace environment where different generations work closely together. While intergenerational coexistence may include generations that are relatively close in age, intergenerational conflicts tend to arise more when age differences are perceived as salient [54]. This generational threshold was chosen to maximize intergenerational contrast and explore salient dynamics or forms of cooperation between age groups while still allowing participants to reflect on their interactions with younger colleagues from a range of age groups. Furthermore, by highlighting the experiences and perspectives of older workers, we sought to better understand the barriers they face as well as the solutions they themselves propose to support their inclusion and healthy participation. There are no exclusion criteria. Since 56% of restaurant workers are women and 44% are men [55], we aim for gender diversity that reflects the industry’s reality.

Participants are recruited in collaboration with the Conseil Québécois des Ressources Humaines en Tourisme, a sectoral workforce committee dedicated to the tourism industry, through the dissemination of a recruitment poster on their social media platforms. This poster is also shared via the research team’s own social media channels. Interested participants are invited to contact us by email. Word-of-mouth also assists in the recruitment process. Additionally, door-to-door canvassing in both commercial and noncommercial food service establishments in the Montérégie-Ouest region of Quebec further supports recruitment efforts. To ensure representative diversity within the foodservice sector, recruitment takes place in several cities across Montérégie-Ouest, including both urban and rural settings. Special attention is given to the selection of establishments to include various types of foodservice (fast food, full-service restaurants, and noncommercial food services). To reach individuals with varying employment statuses (full-time, part-time, day, or evening shifts), door-to-door visits are scheduled across a range of time slots, including weekdays, weekends, daytime, and evening hours. Cultural diversity is also sought by selecting establishments located in areas known for their ethnocultural plurality. The research team verifies participants’ eligibility based on the pre-established inclusion criteria via email or phone.

#### Procedure

Stage 1 consists of 4 steps (see stage 1 in Figure 1). The first step involves an initial one-on-one meeting with each participant. Once participants who meet the inclusion criteria are recruited, a time slot is scheduled for a face-to-face meeting based on their availability. Each participant is met individually at a location of their choice. During this meeting, the project is presented, and key concepts related to the study are defined. For example, the concept of ICW is explained to ensure a shared understanding. The study’s objectives and the photo-elicitation method are also discussed. Participants are provided with a disposable camera, and instructions are given to ensure that they are comfortable using it. A cellphone cannot be used to take the photos for reasons of hygiene and ethics. This meeting also serves as an opportunity to complete the information and consent form, as well as the sociodemographic questionnaire. This questionnaire contains questions about participants’ education, cultural or ethnic background, geographic location, and any disabilities. The second step involves photo-elicitation. Each participant is asked to take photos, with the disposable camera, over a 2-week period, of key elements, objects, or moments they believe either promote or hinder ICW. Photos may be taken at the workplace or outdoors. The location is secondary to the meaning the participant wishes to convey. For ethical reasons, no identifiable faces may appear in the photographs. This photo-taking process includes a prephotographic reflection, during which participants consider what they wish to capture, followed by a postphotographic reflection, where they contemplate how they want the image to be interpreted. To support the feasibility of the process, participants are encouraged to focus on a limited number of photos, ideally around 5, for the focus group. The photos are then developed and digitized. Since the photographs are taken using disposable cameras, the research team is responsible for developing and securely storing the materials, ensuring both the security and anonymity of the images. The photos are kept on a protected, secure institutional platform. Any photos containing identifiable features are anonymized through blurring. Verbal consent from the employer to the participant is required to allow photographs to be taken in the workplace, as specified in the information and consent form that the participant reads and signs. Participants are advised that they must adhere to their employer’s policies and directives related to taking photographs in the workplace.

The third step involves the focus group discussions, which are held remotely and scheduled based on each participant’s availability. Each session lasts approximately 120 minutes, as the recommended duration for focus groups is between 90 and 120 minutes, allowing sufficient time to explore the topic while maintaining participant engagement [52]. If the participant is unable to take part, experiences technical difficulties, or does not have access to the necessary technology, an individual interview will be conducted with this person. During the discussion, participants are invited (1) to respond to questions from a semistructured interview guide covering themes related to ICW and (2) to share their thoughts and reactions to the set of photos, reflecting on what these images represent to them. The semistructured interview guide was developed by 2 members (CC and AL) of the research team and validated by the rest of the team to ensure methodological rigor [56] and an intersectoral perspective. In addition, it was pretested with 2 individuals who share similar characteristics to the study participants. The guide includes questions focused on individual, collective, organizational, and societal practices that may promote or hinder intergenerational cooperation. For example: “Tell me about practices that can be implemented by workers, individually, to promote intergenerational cooperation in your workplace.” Photos serve as the primary discussion material, with the guide offering questions related to the images (eg, “What do you think this image illustrates?”) to help participants express their thoughts and reactions.

The final step involves offering participants the opportunity to provide feedback following the preliminary data analysis. This step is optional. The main preliminary findings will be compiled into a nonpersonal document and sent to participants for their feedback, should they choose to provide it. Their feedback will help enhance the credibility of the results by confirming that they accurately reflect participants’ own words [56].

#### Analysis

First, verbatims will be transcribed from the audio and video recordings of the focus group discussions. A thematic analysis strategy will then be applied to identify and classify emerging themes [57]. The data from both focus groups will be coded based on related ideas. Initially, the data will be categorized based on whether they represent individual, collective, organizational, or societal practices. Further categories will be developed to define emerging concepts and the relationships between them. The raw data will be reviewed multiple times and interpreted using mainly an inductive approach. Theme identification and the development of the thematic tree will follow a continuous thematization approach, ensuring rigor and a rich analysis [57]. The thematization will be supported by NVivo software (Lumivero). Two evaluators will initially analyze the data independently. They will then meet to compare results, resolve discrepancies through discussion, and adjust the categorization based on shared interpretations. This collaborative process will strengthen the analytical rigor and minimize interpretive bias [58]. In case of disagreement between the 2 evaluators during the analysis, a third evaluator (AL) will be consulted to reach a final decision. The Government of Canada’s Gender-Based Analysis Plus approach will be used to assess the potential impacts of the identified practices, considering various identity factors [59]. This approach promotes inclusivity through an intersectional lens, considering dimensions such as disability, culture, education, socioeconomic status, sexual orientation, and geographic location [59]. During the thematic analysis, the Gender-Based Analysis Plus approach will be integrated into both coding and interpretation processes. Coding will involve identifying themes that reflect participants’ experiences in relation to identity factors. These themes will then be interpreted by linking them to participants’ sociodemographic profiles. This process will help reveal how these dimensions may influence participants’ perceptions and experiences related to the issue under study.

In summary, stage 1 of this project will generate a concrete list of individual, collective, organizational, and societal practices that promote intergenerational cooperation with a focus on inclusion and the healthy participation of older workers.

To support effective implementation in workplace settings, it is essential to prioritize this list of practices, providing relevant actors with clear guidance on where to begin. Step 2 of the project will therefore focus on enabling the prioritization of the practices identified during the first phase, through a group of researchers and professionals who will validate and rank the list accordingly. The final list will thus be more robust and will have fostered a dialogue between professionals and researchers.

### Stage 2: Prioritization of the Identified Practices of Intergenerational Cooperation

#### Design

The TRIAGE method will be used to complete this stage. The aim of the method is to achieve a group consensus [60,61]. Originally developed in the field of education, the TRIAGE method is now applied in rehabilitation and health care [61,62]. It is particularly well-suited for prioritizing elements among multiple choices [60]. For instance, some researchers have used the TRIAGE method to prioritize elements in a research process related to the needs of caregivers of older adults [63]. The method differs from other consensus techniques, such as the nominal group technique, which focuses more on generating new ideas, and the Delphi method, an iterative process involving several rounds to reach consensus on complex issues [61,64].

#### Participants

In total, 8 individuals with relevant knowledge will be recruited to participate in the TRIAGE process, in accordance with the literature, which recommends 6 to 12 participants [61]. To address potential participant withdrawal, additional potential participants will be identified in advance and may be invited to join if needed, ensuring that group dynamics and diversity are preserved throughout the study. For this study, we will select individuals with specific knowledge in either management or aging. It would be particularly valuable to include those with experience in foodservice management or researchers with knowledge in intergenerational issues. The goal is to gather diverse perspectives while maintaining a sufficiently homogeneous group to support effective group dynamics [61]. Purposive sampling will be used, as participants will be intentionally selected based on their knowledge and skills related to the study subject [61]. The inclusion criteria are as follows: (1) being a researcher, manager, or professional and (2) having relevant knowledge in management or aging.

Efforts will be made to ensure a diverse and complementary representation of individuals, including both those with knowledge of aging and those with experience in management.

Considering the range of target groups in this study, multiple recruitment strategies will be used:

- For the recruitment of researchers: Potential candidates will be identified by reviewing scientific publications related to management and aging. An email, including a recruitment poster, will be sent to individuals with relevant academic knowledge. Word-of-mouth will also help identify researchers through academic networks. Participants may suggest other individuals with similar experience [60].
- For the recruitment of managers and professionals: A recruitment poster will be shared via the research team’s social media platforms and in online communities targeting foodservice professionals. Word-of-mouth will further support the recruitment of professionals within the community. Additionally, foodservice managers will be approached directly through an outreach strategy such as door-to-door canvassing.

#### Procedure

The TRIAGE method consists of 4 phases (see stage 2 in Figure 1) [61]. The first phase, known as “preparation,” is the longest for the research team [61]. During this phase, the research question to be explored is defined. In this study, this question could be formulated as follows: “Which intergenerational cooperation practices should be prioritized in the foodservice sector?” This phase also involves participant recruitment. To support this process, 3 key documents will be prepared: a cover letter, a preparatory document, and a questionnaire [61]. The cover letter will provide general information about the study and its procedure. The preparatory document will support participants’ reflection by outlining the study’s objectives, defining intergenerational cooperation, and presenting an overview of the TRIAGE method [61]. Finally, the questionnaire, designed to be completed by participants, will serve as the basis for the group session. It will be divided into 2 sections. In the first section, participants will review the practices identified in stage 1, organized into 4 categories: individual, collective, organizational, and societal. Participants will be asked to read all practices listed within a category, rate each one on a scale from 1=priority practice to 5=nonrelevant or unrealistic, and justify their choices. Based on this reflection, they will then rank the practices within each category in order of priority. In the second section, participants will identify the 3 most important practices overall, regardless of category, and may also propose additional practices they believe should be considered. These documents will be pretested with 2 individuals who share similar characteristics to the participants [61]. Once finalized, they will be sent out to participants to initiate the second phase [61].

The second phase, known as “individual production” [61], will be conducted remotely. During this phase, participants will receive and review the preparatory documents. After reading the materials, they will be asked to reflect individually on the questions posed and provide answers based on their experience, as outlined earlier [60]. The aim is to collect participants’ individual perspectives on the proposed practices and to determine which ones they consider most important for prioritization in the foodservice sector.

The third phase, known as “compilation” [61], will begin once the questionnaires have been completed by the participants. The research team will collect and transcribe the responses onto cards [61]. These answers will be transcribed verbatim to accurately reflect the participants’ input, allowing them to recognize their own answers, even though the cards will remain anonymous [61]. To ensure anonymity and improve organization, the cards will be arranged alphabetically [61]. All responses will be included, even those with noticeable repetition [61].

**Figure 2:**
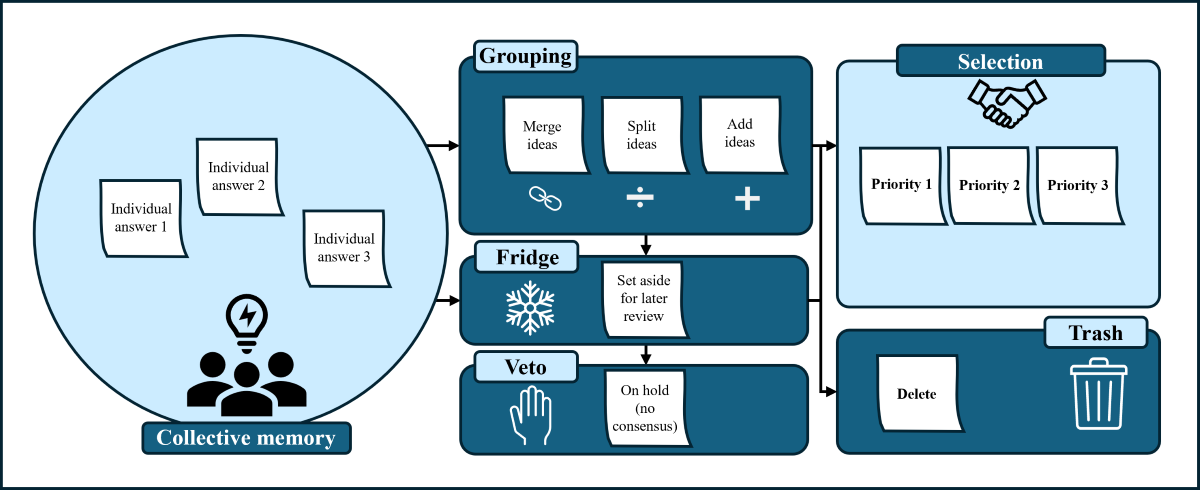
Visual representation of the TRIAGE process. TRIAGE: Technique for Research of Information by Animation of a Group of Experts.

The fourth phase, referred to as “interactive production” [62] or the “group production phase” [60], involves an in-person meeting with all participants. This phase is typically conducted face-to-face; however, in cases of logistical constraints (eg, geographic distance), it may be held virtually using a shared interactive board, as illustrated in the work of Porro et al [65]. The session will last between 2 and 3 hours to allow enough time for discussion while maintaining participants’ attention [61]. The goal of this meeting is to reach a consensus on the prioritization of the intergenerational cooperation practices identified earlier [62]. If the session is held in person, participants will sit around a table facing a board that displays all the individual answers collected on cards [61,62]. Figure 2 shows the different sections of the board that will be presented to participants (collective memory, selection, trash, fridge, veto, and grouping), where individual answers will be discussed. The cards will be presented in the “collective memory” section on the board [61]. Participants will decide, by consensus, whether to keep a practice (“selection” section) or discard it (“trash” section) if there is agreement [61]. The “grouping” section, for its part, allows them to cluster similar ideas, divide or refine them, or add new ideas that emerge during the discussions. If consensus cannot be reached on a specific practice, it may be set aside temporarily (“fridge” section) to be revisited later or completely discarded (“veto” section) if no agreement is reached [61]. Thus, the objective will be to place all the practices in a priority order in the “selection” section based on the consensus of the participants. To ensure balanced participation during the focus groups, 2 facilitators will be present: one will guide the discussion, while the other will monitor speaking time to promote equitable contributions and actively encourage input from quieter participants, thus mitigating the risk of dominance by any subgroup.

As this study uses the TRIAGE method, no scoring or ranking system is applied. The method relies on group discussions and the consensus reached among participants rather than on numerical evaluation. Practices placed in the “selection” section reflect this consensus, emphasizing collective agreement. This shared agreement thus represents the operational consensus threshold for this process.

#### Analysis

Conducting an initial content analysis of the responses from the individual production phase will help identify similarities and divergences [60]. In addition, this method allows for relatively quick data analysis, as the prioritization of practices will be validated by the participants through the consensus reached during the discussions [60,61]. Consequently, results can be accessed immediately after the discussion, provided consensus is reached, by transcribing the answers from the “selection” section of the board.

### Ethical Considerations

Our study was approved by the research ethics committee on rehabilitation and social integration of the Centre Intégré Universitaire de Santé et de Services Sociaux de la Capitale-Nationale (approval: 2025-32-58) as well as by the research ethics committee for human research of the Université du Québec à Trois-Rivières (approval: CER-25-317-10.02) on January 14, 2025. The information and consent form are presented and explained to participants, who then complete it to provide informed consent. Participants are reminded that they may withdraw from the study at any time. All data collected in this study are coded to ensure the confidentiality of participants’ information. They receive compensation of CAN $100 (approximately US $71) for the time they dedicate to the research project.

## Results

Our project has been funded by the Quebec Network for Research on Aging since September 1, 2024. Participant recruitment began in late January 2025, following ethics committee approval. Recruitment of the 12 participants for stage 1 of the study was completed in May 2025. Data analysis is currently underway. Results for this stage are expected to be submitted for publication in 2026.

## Discussion

### Anticipated Impacts of the Research Project

On a theoretical level, this project will provide a prioritized list of practices that promote ICW, with the aim of supporting the inclusion and healthy participation of older workers in the foodservice sector. This list will contribute to advancing knowledge on such practices and will be categorized according to the actors involved. The results may also serve as a foundation for further research, particularly on the impacts of implementing these practices into the foodservice sector, for instance.

On a practical level, this knowledge could support implementation efforts within workplaces, thereby influencing the health and well-being of older workers. Integrating these practices may also promote the retention of older workers by reducing age-related biases and stereotypes, improving the work climate, and minimizing intergenerational conflicts. In turn, enhancing the participation of older workers in the workforce could help address labor shortages, yielding benefits from a societal perspective as well. Given the ongoing labor shortage, exploring such solutions is essential, and mobilizing the older workforce is a strategy currently being considered and applied within the foodservice sector [9].

### Giving a Voice to Older Workers

On one hand, this study will shed light on the perspectives of older workers, contributing to a deeper understanding of intergenerational dynamics in the foodservice sector. Given that few, if any, studies have specifically explored their point of view on this topic, the first stage of the project will offer these individuals an opportunity to express their experiences. The use of photo-elicitation further supports this aim by engaging participants who might not typically take part in traditional question-based research, thus representing a meaningful form of inclusion. In doing so, this study will expand our understanding of the practices that either facilitate or hinder intergenerational cooperation at different levels (individual, collective, organizational, and societal) based on participants’ lived experiences. The photo-elicitation method also promotes active engagement in the research process [51,66] and enhances reflexivity by stimulating more cognitive domains than verbal discussion alone [67]. Additionally, participants’ pre- and postphotographic reflections will enrich the dialogue during the focus groups.

### Adding the Perspective of Individuals With Relevant Knowledge

The TRIAGE method gathers insights from a diverse group of individuals with substantial experience and knowledge in management or aging. Building on the practices identified by older workers, these participants will engage in group discussions to determine which practices should be prioritized in the foodservice sector, drawing on their knowledge. These discussions will foster rich exchanges and support consensus-building, ensuring that the final list of practices is both practical and aligned with the realities and specific needs of the foodservice environment.

### Limitations

The results of the first stage of this study will be based on the photo-elicitation method, which involves participants independently taking photographs over a 2-week period using a provided disposable camera. However, certain limitations may arise if the camera is lost or damaged or if participants forget to take photos during the designated time frame. In such cases, participants will still participate in the focus group, drawing on their own reflections and photographs taken by other participants. In addition, since focus groups require participants to meet at the same time, they will be conducted online to reduce geographical barriers. However, this approach assumes that participants have access to a computer or smartphone and feel comfortable using this format. In addition, door-to-door recruitment may introduce a bias in terms of the representativeness of the diversity within the foodservice sector, as it involves selecting a specific region, which limits the ability to reflect the realities of all regions across Quebec. Furthermore, the absence of younger workers’ perspectives represents a limitation, as it restricts the scope of our conclusions to the experiences of older workers. It would be valuable in future research to carry out a similar exploration with younger workers.

The results of the second stage of this study will be based on the TRIAGE method. Like any method, TRIAGE has both strengths and limitations. Its effectiveness depends on the facilitator’s ability to guide the group discussion and the participants’ perspectives [61]. Additionally, the presence of all participants during the interactive production phase is essential, which can be challenging, particularly due to geographical constraints. In such cases, a virtual format will be used [65]. Moreover, if a participant completes the individual production but is unable to attend the interactive production, this may pose difficulties, as the facilitator and other participants could struggle to fully grasp the nuances of that participant’s contributions presented on the cards.

### Conclusions

Given the aging population and the ongoing labor shortage, it is essential to explore practices that promote the inclusion and retention of older workers in the labor market. In today’s multigenerational workforce, attention must be paid to the impact of intergenerational conflicts on the well-being and health of older workers while also considering the potential benefits of generational diversity, particularly when collaboration is effective. The foodservice sector, which has been especially affected by labor shortages in recent years and is experiencing increased intergenerational coexistence, provides a particularly relevant context for addressing these issues. Therefore, the prioritized list of practices promoting intergenerational cooperation that this project seeks to develop represents an important step in this direction.

## Abbreviations

ICW: intergenerational cooperation at work
TRIAGE: Technique for Research of Information by Animation of a Group of Experts
